# Evidence of Filamin A loss of solubility at the prodromal stage of neuropathologically-defined Alzheimer’s disease

**DOI:** 10.3389/fnagi.2022.1038343

**Published:** 2022-11-24

**Authors:** Etienne Aumont, Cyntia Tremblay, Stéphanie Levert, David A. Bennett, Frédéric Calon, Nicole Leclerc

**Affiliations:** ^1^Département de psychologie de l’Université du Québec à Montréal, Montréal, QC, Canada; ^2^Centre de recherche du Centre hospitalier de l'Université de Montréal, Montréal, QC, Canada; ^3^Montreal Neurological Institute, Montréal, QC, Canada; ^4^Faculté de pharmacie de l’Université Laval, Québec, QC, Canada; ^5^Centre de recherche du Centre hospitalier de l'Université Laval, Québec, QC, Canada; ^6^Département de neurosciences Université de Montréal, Montréal, QC, Canada; ^7^Rush Alzheimer’s Disease Center, Chicago, IL, United States

**Keywords:** Filamin A, Alzheimer’s disease, post-mortem, taupathy, neuropathology

## Abstract

**Introduction:**

Alzheimer’s disease (AD) is a multifactorial disorder diagnosed through the assessment of amyloid-beta (Aβ) and tau protein depositions. Filamin A (FLNA) could be a key partner of both Aβ and tau pathological processes and may be an important contributor to AD progression. The main aim of this study was to describe the differences in FLNA levels across clinicopathologic groups.

**Methods:**

From parietal cortex samples of 57 individuals (19 with no cognitive impairment (NCI), 19 mild cognitively impaired (MCI) and 19 with dementia) from the Religious Orders Study (ROS), we quantified total tau, phosphorylated tau (pTau), FLNA, synaptophysin, vesicular acetylcholine transporters (VAChT) and choline acetyltransferase (ChAT) by Western blot. Aβ42 and neuritic plaques (NP) were quantified by ELISA and Bielschowsky silver impregnation, respectively. AD staging was determined using ABC method combining Thal, Braak and the CERAD staging. From this, clinicopathologic stages of AD were established by subdividing subjects with neuropathological AD between preclinical AD, prodromal AD and AD dementia (ADD). Receiver operating characteristics analyses were performed to predict AD neuropathology from FLNA quantifications.

**Results:**

Insoluble FLNA was significantly and positively correlated with Aβ42, NP, Thal stages, ABC scores and AD clinicopathologic stages (*p* < 0.05 False discovery rate-corrected). No correlation of FLNA with tau measures was found. Insoluble FLNA levels were significantly higher in the prodromal AD, ADD and intermediate ABC groups. This was consistent with significantly lower levels of soluble FLNA specifically in prodromal AD. Insoluble (AUC: 0.830) and soluble FLNA levels (AUC: 0.830) as well as the ratio of soluble over insoluble FLNA (AUC: 0.852), were excellent predictors of prodromal AD among subjects with MCI from the ROS cohort.

**Discussion:**

We observed opposite level changes between insoluble and soluble FLNA in prodromal AD. As this stage coincides with the appearance of cognitive symptoms, this may be a key event in the transition from preclinical to prodromal AD. Insoluble FLNA could be useful to identify prodromal AD among subjects with an MCI, indicating that it might be a hallmark of prodromal AD.

## Introduction

Alzheimer’s disease (AD) is the most frequent cause of dementia, with an estimated prevalence in the United States close to 6 million (de Pedro-Cuesta et al., 2009; Rajan et al., 2021). The neuropathological hallmarks of AD are the presence of neuritic plaques (NP) and paired helical filaments (McKhann et al., 1984; Jack et al., 2018). The former are largely composed of polymerized Amyloid-beta (Aβ) peptides, while the latter are primarily made of hyperphosphorylated microtubule-associated protein tau assembled into paired helical filaments (PHF; Glenner and Wong, 1984; Grundke-Iqbal et al., 1986). These Aβ and tau aggregates are associated with a gradual synaptic loss, which leads to cognitive impairment (Coleman and Yao, 2003; Scheff et al., 2006; Maass et al., 2018; Vanhaute et al., 2020).

AD is now recognized as a multifactorial disease, with Aβ and tau neuropathologies being its most studied lesions (Herrup, 2015; Gong et al., 2018; Samanta et al., 2022). However, other abnormal processes may be more important for identifying, monitoring and predicting the progression, and curing AD. One such promising abnormal process may involve FLNA, which binds to actin filaments and cellular membrane proteins. FLNA is needed for cell structure and function (Zhou et al., 2021). Abnormal function of this protein is thought to contribute to AD neuropathology by contributing to both tau and Aβ pathologies. As a first hint of its malfunction in AD, FLNA was found to colocalize with PHFs (Feuillette et al., 2010). It was then found that Aβ, mostly in the form of Aβ_42_, structurally altered FLNA in AD (Burns and Wang, 2017). This alteration allowed FLNA to bind to α7 nicotinic acetylcholine receptors (α7nAChR) and toll-like receptor-4 (TLR4), altering their response (Wang et al., 2012, 2017; Burns and Wang, 2017). Aβ_42_ then interacts with the FLNA-bound α7nAChR and TLR4 to induce neuroinflammation and tau hyperphosphorylation, thereby contributing to disease progression in animal models (Burns and Wang, 2017). Reversing FLNA structural alteration using the compound PTI-125 reduced tau, neuroinflammation and Aβ biomarkers in a phase IIa clinical trial (Wang et al., 2020). FLNA may also contribute to tau pathology in the absence of Aβ. From cell culture experiments, Tsujikawa et al. (2022) reported that higher FLNA levels increased tau protein levels, phosphorylation and insolubility, while its reduction was shown to decrease these effects on tau. Collectively the above observations indicate that the abnormal function of FLNA could be crucial for inducing the neuropathological processes of AD. However, our knowledge of *in vivo* alterations of FLNA across the stages of AD remains elusive.

The primary aim of the present study was to describe the FLNA levels over the different stages of AD. A secondary aim was to identify which AD-associated features were predicted by FLNA levels. To do so, we analyzed post-mortem cortex samples and AD staging data obtained from the Religious Orders Study. FLNA, NP, Aβ_42_, total tau and phosphorylated tau (pTau) levels were quantified by immunoblotting or ELISA. We used FLNA levels to predict Aβ and tau-derived sample levels. We then assessed the predictive power of FLNA over general disease progression stages in the form of increased insoluble FLNA levels at later stages of AD.

## Materials and methods

### Participants

Samples from the precuneus cortex (Brodmann’s Area 7) were obtained from 60 participants in the Religious Orders Study (Bennett et al., 2012). The study was approved by an Institutional Review Board of Rush University Medical Center. All subjects signed informed consent, Anatomic Gift Act, and repository consent. The subjects were distributed across three age-matched cognitive profiles: no cognitive impairment (NCI, N = 20), mild cognitively impairment (MCI, N = 20) and dementia (N = 20), based on a clinical evaluation that was previously described (Bennett et al., 2002, 2006). In short, these diagnoses were based on a yearly medical visit including a mini-mental state examination (MMSE) as well as a standardized neuropsychological battery of 19 standardized cognitive performance indicators from five cognitive domains, which were, in turn, integrated into composite scores (Bennett et al., 2012; Wilson et al., 2015). Three participants – one from each cognitive profile group - were excluded from the study due to missing data.

At death, each case was assessed using the revised National Institute on Aging–Alzheimer’s Association (NIA-AA) guidelines for the neuropathologic assessment of Alzheimer’s disease (Montine et al., 2012). This includes the assessment of Aβ deposits with Thal’s stages (A; Thal et al., 2002), that of PHF with Braak stages (B; Braak and Braak, 1991) and that of NP distribution with the Consortium to Establish a Registry for Alzheimer’s Disease (CERAD) scores (C; Mirra et al., 1991). Using the chart from the revised NIA-AA guidelines, four levels ABC scores, representing levels of AD neuropathological changes were obtained: “not,” “low,” “intermediate” or “high.” To avoid any confusion between clinical and pathological nosological definitions, all mentions of AD in this work refer to intermediate or high levels of AD neuropathological changes. Intermediate or high levels of AD neuropathological changes are consistent with a neuropathological diagnosis of AD, as one needed to display at least intermediate scores on both Braak (stage III) and one of the Aβ subscales (either Thal stage 3 OR Thal stage 1 with moderate CERAD assessment). This is opposed to individuals with not or low levels of AD neuropathological changes, for whom AD neuropathology is insufficient to account for any cognitive symptom (N = 20; 10 NCI, 8 MCI and 2 with dementia; Montine et al., 2012). We further subdivided the AD group into three clinicopathologic stages based on the antemortem clinical groups: preclinical AD (N = 9; all NCI), prodromal AD (N = 11; all with MCI) and AD dementia (ADD; N = 17; all with dementia). These groups are described in Table 1. Notably, subjects with ADD had significant cognitive deficits when compared to all other groups. As anticipated from previous studies, non-AD subjects had a significantly lower frequency of Apolipoprotein E allele *ε4* (APOE ε4) carriers (Poirier et al., 1993; Bennett et al., 2003).

**Table 1:** Characteristics of subjects classified by clinicopathologic stages of AD.

Characteristics	Non-AD	Preclinical AD	Prodromal AD	AD dementia	Statistical analysis
N	20	9	11	17	n/a
Age	86.5 (4.43)	87.1 (7.24)	88.6 (5.71)	87.1 (5.26)	*F*(53) = 0.36; *p* = 0.78
Men (%)	45	11.1	36.4	29.4	*χ*^2^(3) = 3.39; *p* = 0.34
APOE ε4 carriers (%)	10^*^	55.6	45.5	41.2	*χ*^2^(3) = 8.10; p = 0.04
Years of education	18.5 (3.61)	18.7 (4.12)	17.5 (3.24)	18.1 (2.38)	*F*(53) = 0.29; *p* = 0.83
MMSE score	25.6 (4.15)	27.2 (1.56)	25.1 (2.98)	15.8 (8.59)^*^	*F*(53) = 13.3; *p* < 0.0001
Clinical diagnosis (NCI/MCI/Dementia)	10/8/2	9/0/0	0/11/0	0/0/17	
Postmortem interval, hours	7.14 (4.66)	7.73 (6.87)	8.67 (5.70)	6.99 (4.12)	*F*(51) = 0.27; *p* = 0.85
Cerebellar pH	6.40 (0.33)	6.25 (0.33)	6.29 (0.27)	6.31 (0.44)	*F*(53) = 0.46; *p* = 0.71
Prevalence of infarcts (%)	30	22.2	27.3	17.6	*χ*^2^(3) = 0.83; *p* = 0.84
Prevalence of CAA (%)	25	44.4	44.5	47.1	*χ*^2^(3) = 3.06; *p* = 0.38
pS409/410 insoluble TDP-43	7.63 (5.58)	6.40 (4.80)	10.7 (5.01)	9.66 (4.97)	*F*(53) = 1.53; *p* = 0.22
pS409/410 soluble TDP-43	97.0 (25.6)	100 (28.6)	96.9 (16.7)	84.8 (15.4)	*F*(53) = 1.41; *p* = 0.25
Thal stages 0/1/2/3/4/5 (N)	7/11/2/2/0/0	0/2/0/3/1/3	0/0/0/7/3/2	0/1/0/5/4/7	
Braak stages I/II/III/IV/V (N)	3/4/9/6/0	0/0/3/6/0	0/0/4/7/1	0/0/5/2/10	
CERAD stages 4/3/2/1 (N)	14/4/4/0	0/1/6/2	1/1/6/4	0/1/4/12	
Episodic memory CS	−0.05 (0.87)	0.04 (0.35)	−0.42 (0.62)	−1.97 (1.17)^*^	*F*(53) = 17.74; *p* < 0.0001
Semantic memory CS	−0.21 (0.76)	−0.28 (0.35)	−0.52 (0.60)	−1.48 (1.22)^*^	*F*(53) = 7.63; *p* = 0.0002
Working memory CS	−0.26 (0.44)	−0.15 (0.58)	−0.71 (0.69)	−0.94 (0.92)^2^	*F*(53) = 4.30; *p* = 0.009
Processing speed CS	−0.77 (1.08)	−0.21 (0.78)	−0.94 (0.71)	−2.10 (0.87)^*^	*F*(53) = 10.67; *p* < *p* < 0.0001
Visuospatial ability CS	−0.10 (0.76)	−0.41 (0.56)	−0.24 (0.68)	−1.06 (0.98)^3^	*F*(53) = 4.93; *p* = 0.004
General cognition CS	−0.20 (0.57)	−0.11 (0.30)	−0.52 (0.38)	−1.65 (0.87)^*^	*F*(53) = 20.5; *p* < 0.0001

Non-AD: either “not” or “low” score of neuropathological change on the ABC scale; AD: either “intermediate” or “high” score on the ABC scale; Preclinical AD: no cognitive impairment along with AD; Prodromal AD: AD neuropathology in the presence of a mild cognitive impairment; CAA: cerebral amyloid angiopathy; TDP-43: transactive response DNA binding protein 43; ^*^p < 0.05 versus all other groups; ^2^*p* < 0.05 versus Non-AD and Preclinical AD; ^3^*p* < 0.05 versus Non-AD and Prodromal AD.

### Preparation of brain homogenates

The cortical samples (~100 mg) were sequentially homogenized, sonicated and centrifuged. The first extraction was performed in tris-buffered saline (TBS) to generate a TBS-soluble fraction, containing mostly cytosolic and extracellular proteins. The second extraction was performed in detergents (0.5% sodium deoxycholate, 0.5% sodium dodecyl sulfate and 1% Triton X-100) to extract membrane-bound proteins (i.e., detergent-soluble fraction). Lastly, the detergent-insoluble fraction containing proteins from insoluble aggregates was extracted through solubilization in formic acid. A detailed method of the protein extraction was previously described (Tremblay et al., 2007, 2017; Julien et al., 2008, 2009; Bourassa et al., 2019, 2020).

### Western blot and ELISA

For Western immunoblotting, 15 μg of total proteins per lane, as measured using bicinchoninic acid assays (Pierce/Thermo Fisher Scientific, Waltham, MA) were added to Laemmli’s loading buffer. The solution was heated to 95°C for 5 min, then subjected to sodium dodecyl sulfate polyacrylamide gel electrophoresis. Proteins were electroblotted onto PVDF membranes (GE Healthcare, Mississauga, ON, Canada). The membranes were then blocked in 5% non-fat dry milk and 0.5% bovine serum albumin in 10 mM phosphate-buffered saline for 1 h before being immunoblotted with the appropriate primary and secondary antibodies. Bands were visualized by chemiluminescence (Luminata Forte, Millipore, Etobicoke, ON, Canada), then acquired and analyzed with an Amersham imager 680 (GE healthcare). Relative optical density values of TBS-soluble proteins were normalized to actin. In addition, insoluble FLNA was normalized to sample weight, due to the absence of reliable housekeeping proteins in the formic acid fraction. The following antibodies were used: anti-actin (ABM, Richmond, BC, Canada), anti-FLNA (#4762, Cell Signaling Technology, Danvers, MA, United States), anti-total Tau (OST00329W, epitope: 323–363 a.a., Osences, SA, Australia), anti-pTau (PHF-1, epitope: pS396/404 gift from Dr. Peter Davies; Feinstein Institute for Medical Research, Manhasset, NY), anti-synaptophysin (SVP-38, Millipore), anti-VAChT (OSV00002G, Pierce/Thermo Fisher Scientific), anti-ChAT (Proteintech, Rosemont, IL) and anti-phosphorylated transactive response DNA binding protein 43 (pTDP-43, epitope: pS409/S410, Lumiglo Reserve). Detergent-soluble fractions were used for quantification of synaptophysin, VAChT and ChAT, while detergent-insoluble fractions were used for total Tau, pTau and FLNA. In addition, TBS-soluble FLNA quantification was obtained. All of these analyses were performed in two batches.

Concentrations of Aβ_42_ were determined in the FA-soluble fraction, which was resuspended in guanidine, using high sensitivity ELISA (Wako, Japan), as described elsewhere (Tremblay et al., 2007). Plates were read at 450 nm using a SynergyTM HT multi-detection microplate reader (Biotek, Winooski, VT).

Neuritic plaques were counted following Bielschowsky silver impregnation from parietal cortex samples (See Bennett et al., 2003 for more details). A logarithmic transformation was applied on the NP count in order to adjust for the asymmetry of the distribution.

### Statistical analyses

All statistical analyses were performed using RStudio version 2021.9.1.372 (RStudio Team, 2020) using a base statistical significance threshold of two-tailed *p* = 0.05. We performed partial linear correlations between the levels of FLNA and those of Aβ_42_, pTau, total Tau, NP, synaptophysin, VAChT, and ChAT. Age, sex, and study batch were included as covariates in the partial correlations. APOE ε4 carrier status was not used as a covariate due to the small number of non-AD carriers (*N* = 2). To improve the accuracy of data visualization corresponding to the statistical analyses, the figures depict predicted values, which were obtained from the protein level residuals from age, sex and study batch regressions. The initial protein level mean was added to the residuals, resulting in predicted protein levels when removing the age, sex and study batch effects. ABC score, its three subscores (Thal, Braak and CERAD) and AD clinicopathologic stages were considered as ordinal variables for statistical analysis purposes. As such, the association between FLNA levels and ABC scores, subscores and AD clinicopathologic stages were assessed with Spearman’s non-parametric correlations. In these instances, FLNA was sequentially adjusted for age, sex and study batch. Inflation of the false discovery rate (FDR) due to multiple tests was corrected through the adjustment of the *p*-values by using the Benjamini-Hochberg procedure (Benjamini and Hochberg, 1995). Unless otherwise specified, p-values presented in the results section are FDR-corrected, with full results displayed in Supplementary Table 1. Due to small N, Braak stages I and II were merged.

Additional partial linear correlations between Aβ-derived measures (Aβ_42_ concentrations and NP counts) and tau-derived measures (total Tau and pTau levels) were performed to verify the interplay between tau and Aβ neuropathology. Similarly, we performed Spearman’s correlations between Aβ stages (Thal and CERAD stages) and Braak stages. These control analyses were not FDR-corrected.

As a *post-hoc* to the significant Spearman’s correlations, two-tailed Dunnett tests were performed comparing FLNA levels at the least severe AD stage to the one at each of the other stages. This was meant to allow identification of the stages where FLNA levels were significantly different from those found at the earliest stage of AD. Additionally, we performed Spearman’s correlations on Thal and CERAD stages) and tau measures (total Tau, pTau and Braak stages). Adjusted standardized mean differences (SMD) were used as measures of effect sizes in group comparisons.

As a supplementary analysis to verify the effect of APOE ε4, we performed two ANCOVAs comparing insoluble and soluble FLNA in three groups: APOE ε4 noncarriers with AD, APOE ε4 carrier with AD, and subjects without AD. Tukey *post-hoc* tests were conducted to reveal group differences.

Receiver operating characteristics (ROC) analyses were performed to measure the ability of FLNA adjusted for age, sex, APOE ε4 carrier status and study batch to identify the subjects with AD. Three such analyses were performed: (1) to identify AD from the whole sample, (2) to identify prodromal AD from the MCI group and (3) to identify preclinical AD from the NCI group. *p*-values for those results were FDR-corrected as a separate set. The discriminative ability of predictors was interpreted based on Hosmer et al.’s guidelines Hosmer et al. (2013) for the area under the curve (AUC). AUC, sensitivity and specificity values were compared qualitatively to those obtained using Aβ_42_, pTau, total Tau and NP adjusted for age, sex and study batch. Full results for those ROC analyses are displayed in Supplementary Table 2.

Semi-partial linear correlations were performed to verify the association between FLNA levels and cognitive variables, including all five cognitive domain scores, the general cognition domain composite scores and the MMSE score. Cognitive scores were adjusted for age and sex and years of education, while FLNA was adjusted for age, sex and study batch. Due to the different nature of the cognitive variables, FDR correction with the Benjamini-Hochberg procedure was performed independently on the resulting *p*-values. Full results are displayed in Supplementary Table 3.

In a last set of analyses, partial correlations were performed to verify the association between FLNA and phosphorylated soluble and insoluble pTDP-43 levels. The effect of the presence of infarcts and cerebral amyloid angiopathy (CAA) on FLNA was also tested using ANCOVAs. These results were included in an independent set of FDR correction.

## Results

### Insoluble FLNA correlation with Aβ and tau neuropathologies

**Figure 1:**
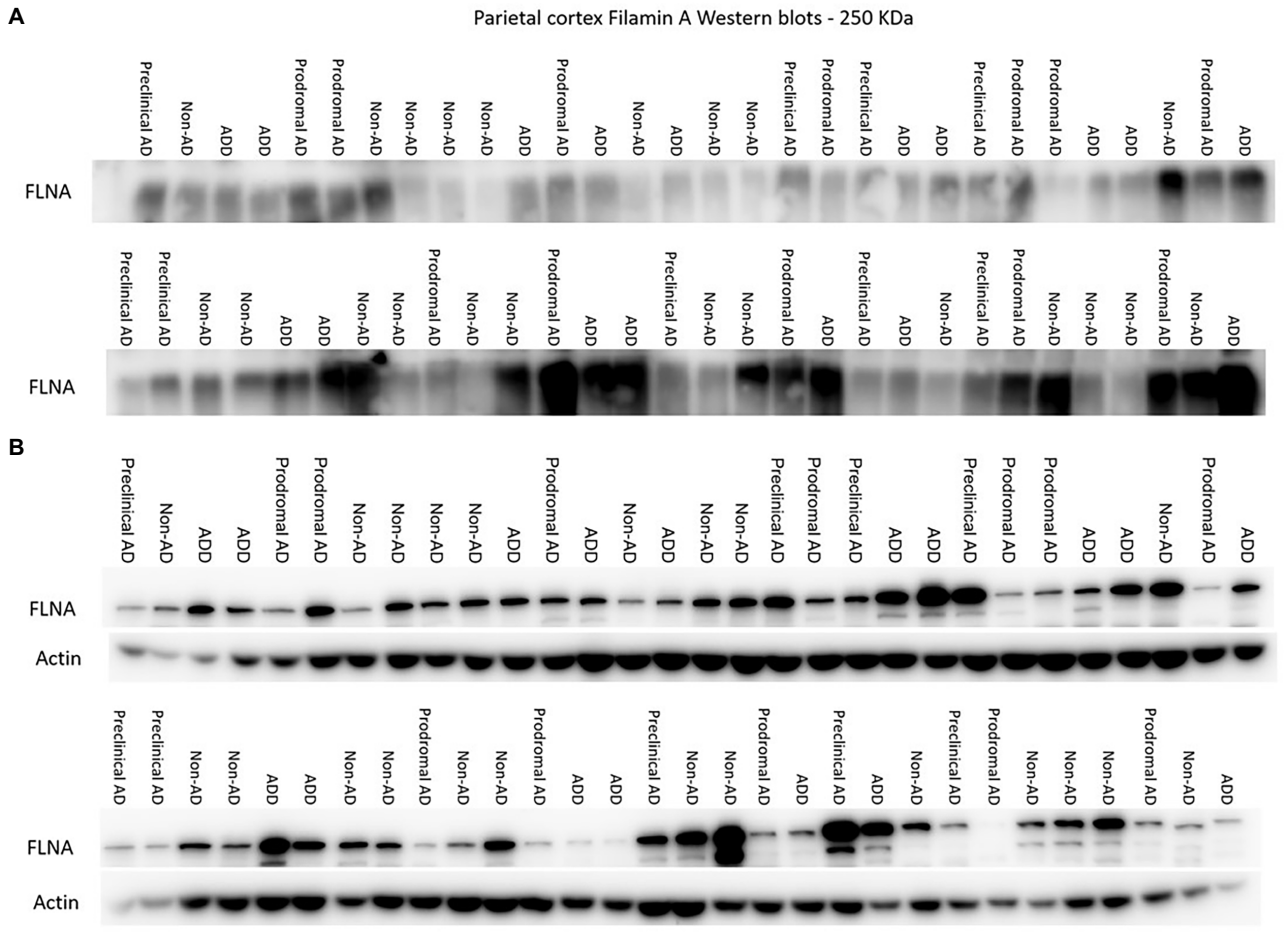
Western immunoblotting of insoluble **(A)** and TBS-soluble **(B)** Filamin A (FLNA) migrating at approximately 250 kDa. Actin (42 kDa) is shown in soluble fractions. Subjects are identified by group. ADD, AD dementia.

**Figure 2:**
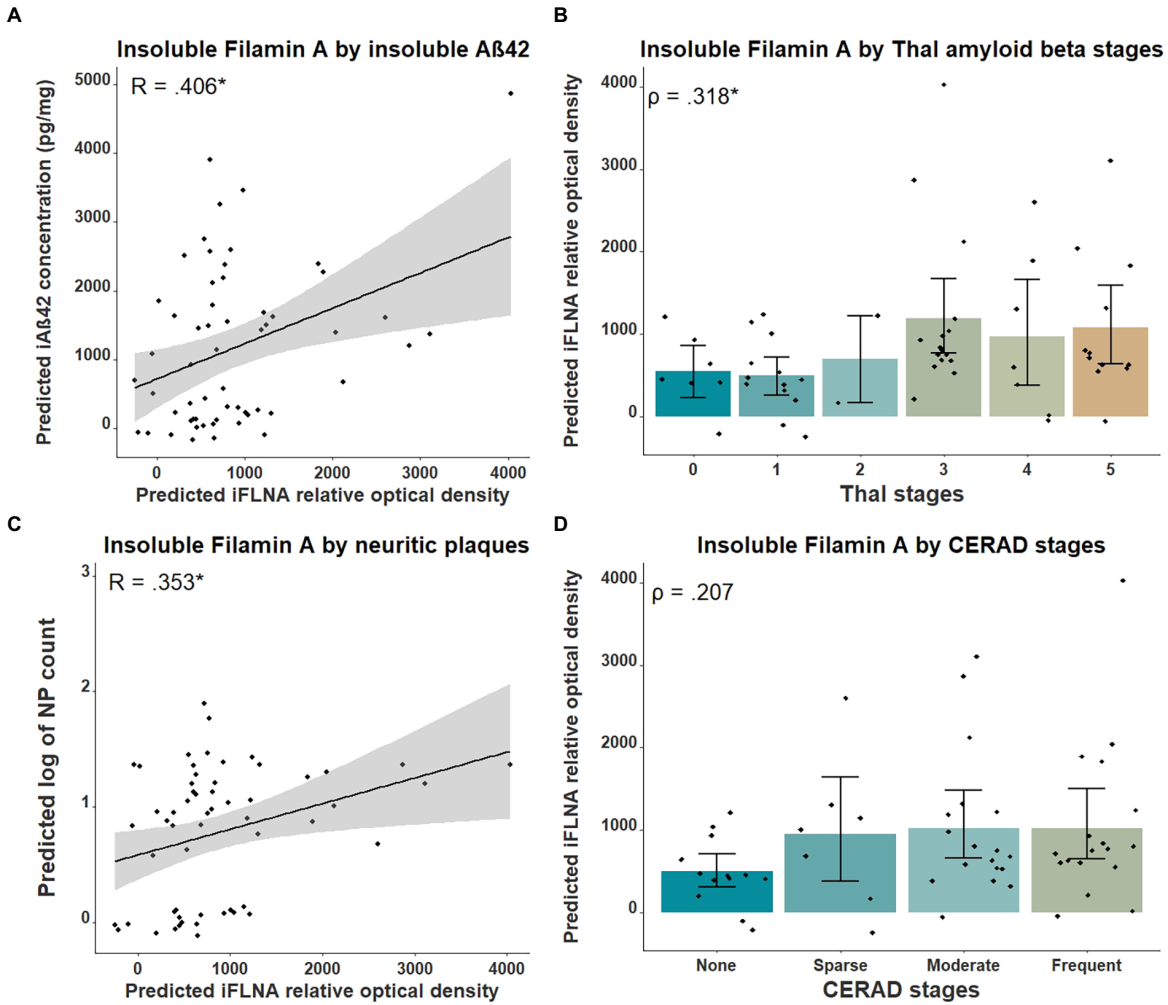
Associations between insoluble FLNA and Aβ-derived metrics. Insoluble FLNA (iFLNA) levels are significantly associated with insoluble Aβ_42_ [iAβ_42_; **(A)**], Thal stages **(B)** and neuritic plaques (NP) **(C)**. However, the association with CERAD stages is not significant **(D)**. Predicted insoluble FLNA, Aβ42 and NP values shown were calculated by adding the mean to the residual values from age, sex and study batch linear regression. Standardized beta and rho values are used to display the strength of associations. *: *p* < 0.05.

Previous studies in cellular and animal models including ours (Levert et al., 2022, in press; Tsujikawa et al., 2022) revealed that upon FLNA overexpression, an increase of tau protein levels and phosphorylation, two events reported in early stages of AD, were observed (Braak et al., 2011). Based on this, we anticipated that FLNA alterations might be associated with tau accumulation and hyperphosphorylation prior to the formation of NFTs. In this context, the parietal cortex, a region affected at late stages of AD seemed ideal for capturing the correlation of the changes of FLNA protein levels and solubility with the progression of Aβ and tau pathologies (Braak et al., 2006). To verify the association between Aβ-associated neuropathology and detergent-insoluble FLNA accumulation, we quantified the levels of insoluble FLNA and Aβ_42_ in homogenates of parietal cortex samples. FLNA levels was determined by western blotting and Aβ_42_ by ELISA (Figure 1). NP count was measured in the same region. We used Thal and CERAD that are based on observations from several brain regions as measures of global neuropathology. We found that FLNA was significantly and positively associated with Aβ_42_ concentrations (β = 0.406, *p* = 0.036; Figure 2A), with Thal stages (ρ = 0.318, *p* = 0.042; Figure 2B), and with NP count (β = 0.353, *p* = 0.042; Figure 2C). CERAD stages were not significantly associated with insoluble FLNA (Figure 2D). None of the Thal stage groups were found to display significantly higher insoluble FLNA levels than Thal stage 0.

**Figure 3:**
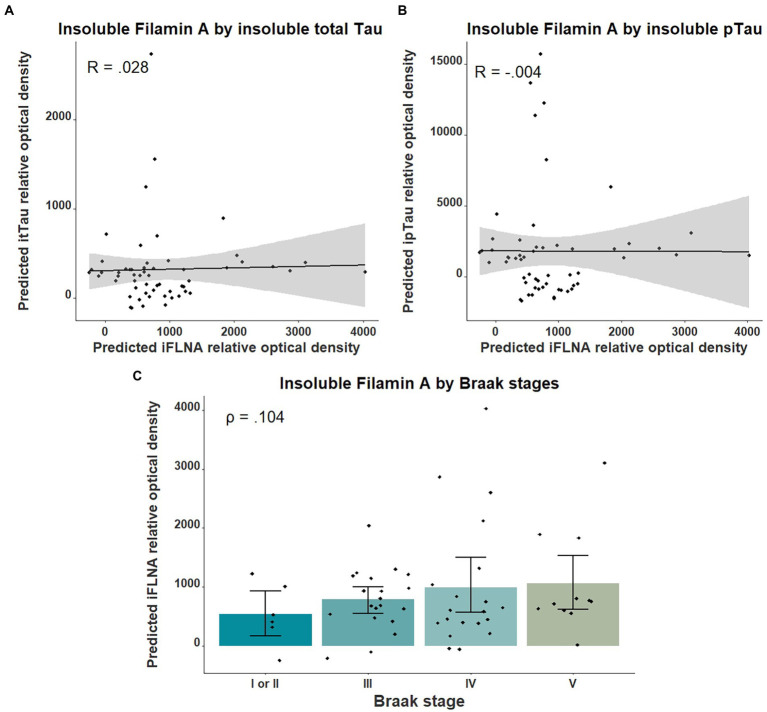
Associations between insoluble FLNA and tau-derived metrics. Insoluble FLNA (iFLNA) levels were not significantly associated with either insoluble total tau [itTau; **(A)**], insoluble phosphorylated tau [ipTau; **(B)**] and Braak stages **(C)**. Predicted insoluble FLNA, itTau, and ipTau values shown were calculated by adding the mean to the residual values from age, sex, and study batch linear regression. Standardized beta and rho values are used to display the strength of associations.

We then investigated the association between insoluble FLNA levels and tau-associated neuropathology using parietal cortex levels of pTau and total Tau as well as the Braak stages. No significant association was found between insoluble FLNA and these tau-derived measures (Figure 3). To ensure the validity of our quantifications of tau, we assessed the levels of total Tau across the ABC scale and Braak stages. This showed a strong significantly higher level at the “high” stage when compared to the “not” stage (*p* = 0.00006, SMD = 1.77; Supplementary Figure 1A). In contrast, “intermediate” and “low” stages had similar total Tau levels to those found at the “not” stage. Similarly, total Tau levels were specifically higher at Braak stage V, but not at earlier stages (Supplementary Figure 1B).

As an additional validation of our AD neuropathology measurements, we tested the association of Aβ-derived metrics with tau-derived metrics. We found NP count to be positively correlated with total Tau (β = 0.494, uncorrected *p* = 0.0003) and pTau (β = 0.524, uncorrected *p* = 0.0001) levels, while Aβ_42_ was positively associated with pTau (β = 0.401, uncorrected *p* = 0.004), but the association with total Tau (β = 0.340, uncorrected *p* = 0.017) did not survive FDR-correction. Similarly, Braak stages were significantly correlated with Thal (ρ = 0.593, uncorrected *p* < 0.0001) and CERAD (ρ = 0.502, uncorrected *p* < 0.0001) stages (Supplementary Figure 2). This confirmed that insoluble FLNA levels were indeed exclusively associated with Aβ neuropathology, but not with tau despite both tau and Aβ neuropathological stages being strongly correlated with one another.

### Association of FLNA with stages of AD

**Figure 4:**
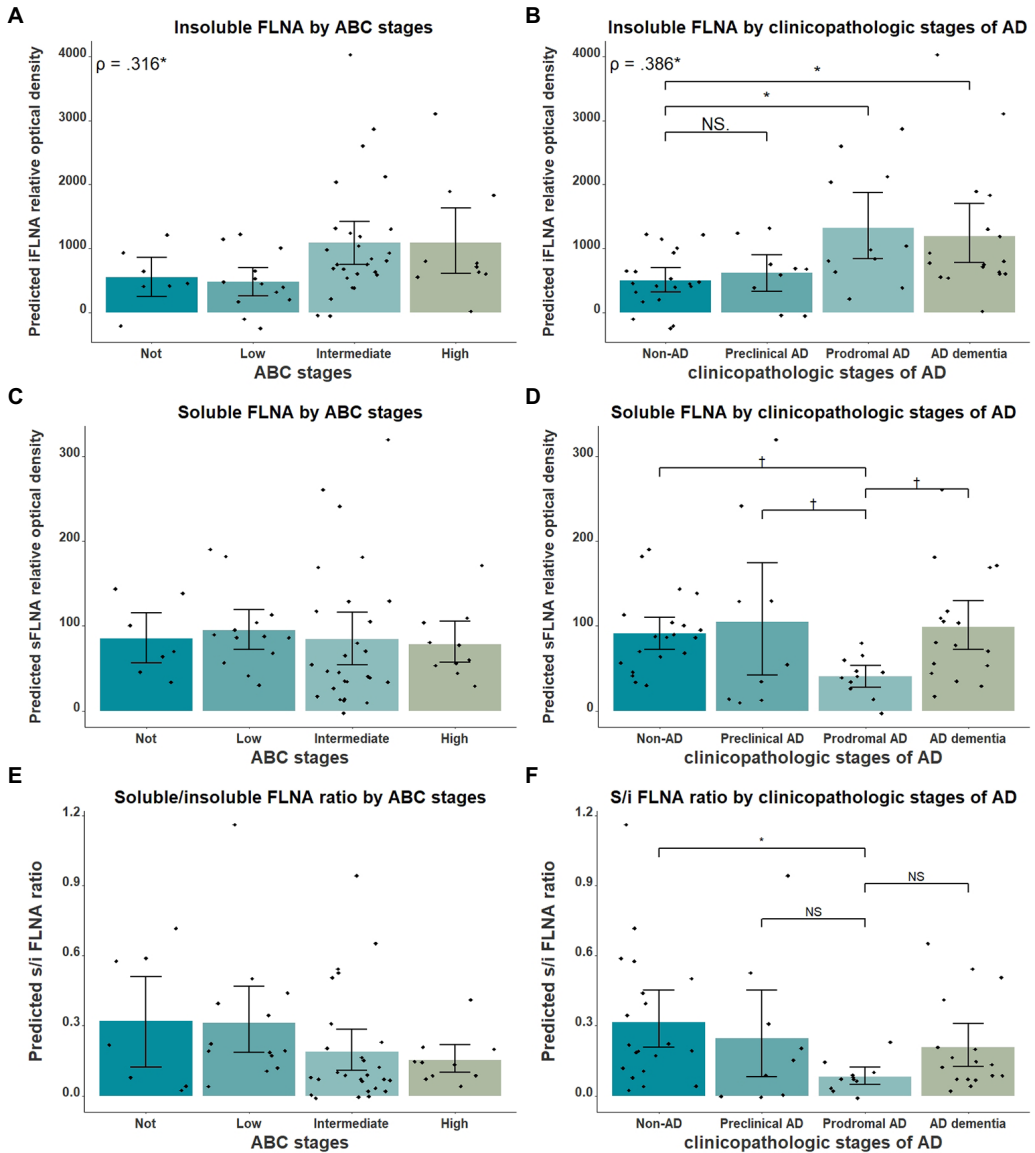
FLNA association with ABC scores and clinicopathologic stages of AD. **(A)** Insoluble FLNA (iFLNA) significantly correlates with ABC scoring, with the “not” score being associated with significantly lower FLNA levels than the “intermediate” score. **(B)** Insoluble FLNA significantly correlates with clinicopathologic stages of AD, with the non-AD displaying significantly lower FLNA levels than the prodromal AD and the ADD. **(C)** Soluble FLNA (sFLNA) levels did not vary across the ABC scale. **(D)** A trend toward lower levels of soluble FLNA at the prodromal AD stage than at any other clinicopathologic stage of AD was found. (**E** and **F**) Correlation of the soluble/insoluble FLNA ratio with the ABC stages and clinicopathological stages of AD. S/I: soluble over insoluble FLNA ratio. Predicted soluble and insoluble FLNA values shown were calculated by adding the mean to the residual values from age, sex and study batch linear regression. Standardized beta and rho values are displayed to the strength of associations. †: *p* < 0.1; *: *p* < 0.05.

Next, we verified the association between insoluble FLNA and the general stages of AD using AD clinicopathologic stages and the ABC scale. Insoluble FLNA levels were positively correlated with the ABC scores (ρ = 0.316, *p* = 0.042; Figure 4A) and AD clinicopathologic stages (ρ = 0.386, *p* = 0.036; Figure 4B). However, no ABC stage was found to have significantly different insoluble FLNA levels when compared to the “not” stage. When compared to the non-AD subjects, the prodromal AD (*p* = 0.037, SMD = 0.94) and the ADD subjects (*p* = 0.036, SMD = 0.80) had significantly higher insoluble FLNA levels (Figure 4B). This indicated that the insoluble FLNA levels might represent a specific aspect of the clinicopathologic progression of AD. This also suggested that a stabilisation of insoluble FLNA levels may take place at later stages of AD, as its levels were similar in both ADD and prodromal AD stages.

We also investigated the relationship between soluble FLNA and the clinicopathologic stages of AD. We expected soluble FLNA to be lower at the prodromal AD stage, where insoluble FLNA levels was significantly increased. Indeed, we found a trend toward lower soluble FLNA levels at the prodromal AD stage when compared to the non-AD (*p* = 0.096, SMD = 0.76), to the preclinical AD (*p* = 0.073, SMD = 0.99) and to the ADD groups (*p* = 0.053, SMD = 0.89; Figure 4D). We calculated the soluble FLNA to insoluble FLNA ratio. We found that this ratio was significantly lower at the prodromal stage when compared to the non-AD (*p* = 0.028, SMD = 0.98), although no difference was found when compared with the other groups (Figure 4F). In contrast, there was no significant difference between both soluble FLNA levels and soluble/insoluble FLNA ratio across ABC scores (Figures 4C,E). Therefore, a decreased soluble FLNA levels mirrored higher insoluble FLNA levels at the prodromal AD stage. In contrast, in ADD, soluble FLNA levels were normal, reflecting the similar levels of insoluble FLNA in ADD and prodromal AD. This was specific to AD clinicopathologic stages, with no differences in soluble FLNA levels across the ABC scale, suggesting that soluble FLNA may be linked to the clinicopathologic stages of AD. The ratio of soluble and insoluble FLNA did not appear to hold any advantage when compared to insoluble FLNA.

**Figure 5:**
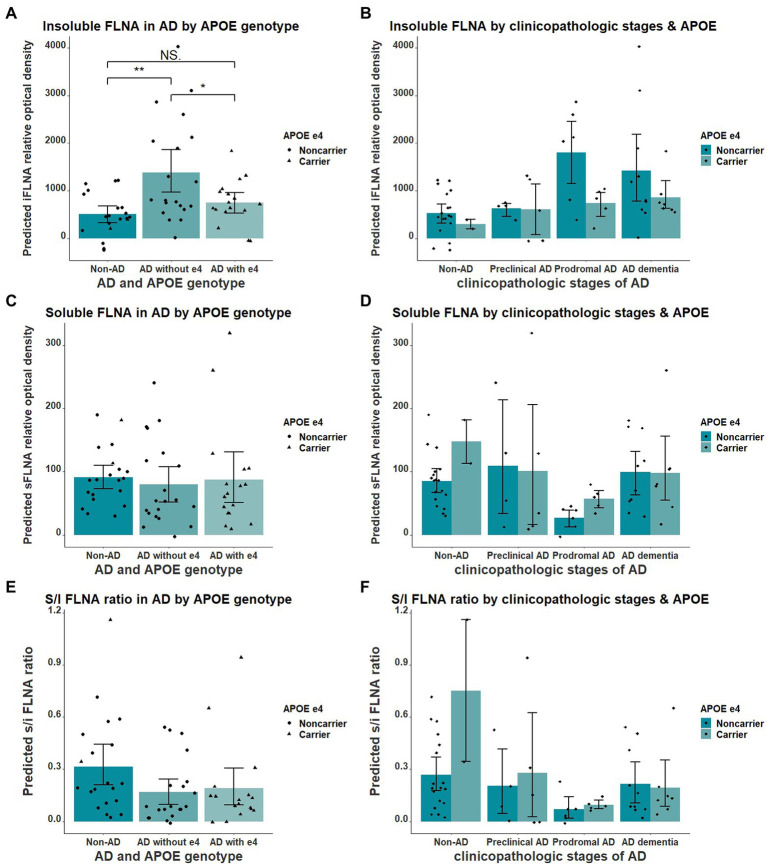
Interaction of APOE ε4 and AD over the levels of FLNA. **(A)** Insoluble FLNA (iFLNA) is significantly increased in participants with AD that do not carry APOE ε4 when compared to AD carriers and non-AD subjects. **(B)** This contrast is most apparent at the prodromal stage of AD. **(C–F)** No significant effect of APOE ε4 on either soluble FLNA (sFLNA) of soluble over insoluble (s/i) FLNA ratio in AD was found. Predicted soluble and insoluble FLNA values shown were calculated by adding the mean to the residual values from age, sex and study batch linear regression. *: *p* < 0.05; **: *p* < 0.01.

We found the APOE ε4 allele to significantly impact the insoluble FLNA levels in AD when comparing participants without AD, ε4 noncarriers AD and ε4 carriers AD participants (*F* = 7.348(54), *p* = 0.002, Figure 5A). Insoluble FLNA levels were significantly higher in AD noncarriers when compared with both subjects without AD (*p* = 0.001, SMD = 1.06) and AD carriers (*p* = 0.034, SMD = 0.76) while FLNA levels did not significantly differ between AD carriers and subjects without AD (*p* = 0.575, SMD = 0.30). Similarly, APOE ε4 carriers with prodromal AD did not display greater FLNA levels (Figure 5B). By contrast, either soluble FLNA and soluble/insoluble FLNA ratio did not significantly differ across groups (Figures 5C-F). Separating subjects by both APOE genotype and clinicopathologic stages of AD hinted at a pronounced increased insoluble FLNA levels of APOE ε4 noncarriers at the prodromal stage (Figure 5D).

**Figure 6:**
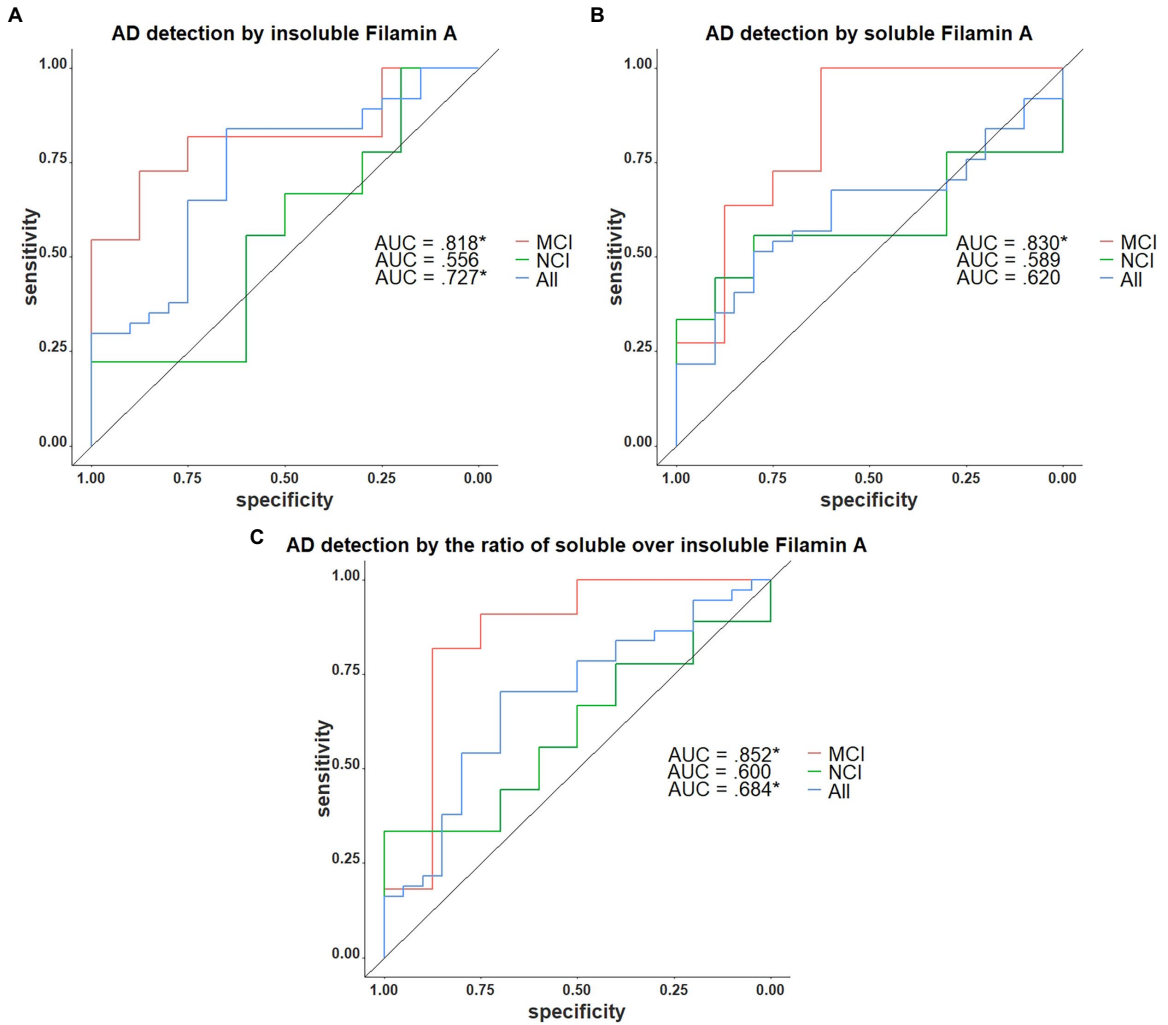
Receiver operating characteristics curves for the identification of AD with FLNA. **(A)** Insoluble FLNA is an adequate predictor of AD in the whole sample, an excellent predictor in the mild cognitively impaired (MCI). Insoluble FLNA is an inefficient predictor in the non-cognitively impaired (NCI). **(B)** Soluble FLNA does not predict AD in the whole sample nor in the NCI. Soluble FLNA is an excellent predictor of AD in the MCI. **(C)** The ratio of soluble over insoluble FLNA is an adequate predictor of AD in the whole sample, an excellent predictor in the MCI, and does not predict AD neuropathology in the NCI. Insoluble, soluble and soluble over insoluble FLNA levels were corrected for age, sex, and study batch. *: *p* < 0.05.

We next assessed the potential of insoluble FLNA levels as a predictor of the AD neuropathological diagnosis. In the present sample, insoluble FLNA was an adequate predictor of AD at any stage (AUC = 0.722, *p* = 0.036) and did not predict preclinical AD among the subjects with NCI (AUC = 0.522, *p* = 0.905). However, when used specifically to identify subjects with prodromal AD among those with an MCI, insoluble FLNA was an excellent predictor (AUC = 0.818, *p* = 0.041; Figure 6A). Similarly, soluble FLNA was an excellent predictor of prodromal AD among subjects with an MCI (AUC = 0.830, *p* = 0.041), but not for the discrimination of preclinical AD among subjects with NCI nor for the discrimination of all-stages AD from the whole sample (Figure 6B). The soluble/insoluble FLNA ratio yielded similar results, being an excellent predictor of AD in the MCI (AUC = 852, *p* = 0.041) and a poor predictor of AD in the whole sample and in the NCI subsample (Figure 6C; see Supplementary Table 2 for full results).

### Association of FLNA with neurodegeneration, cognition, and co-pathologies

Insoluble FLNA did not significantly predict cognitive performance on any of the scales (Supplementary Table 3). By contrast, Aβ_42_, NP, pTau and total Tau were all strong predictors of global cognition and memory, with weaker associations with other cognitive domains. The correlation of insoluble FLNA levels with synaptic markers and cognitive performance was examined. Insoluble FLNA was not found to be correlated with detergent-soluble synaptophysin, a presynaptic vesicular protein whose reduced levels reflect neurodegeneration (Wiedenmann and Franke, 1985). It did not significantly correlate with detergent-soluble VAChT and detergent-soluble ChAT either (Supplementary Table 1, data not shown).

Lastly, we investigated the association of vascular and pTDP-43 co-pathologies with soluble and insoluble FLNA. Neither soluble pTDP-43, insoluble pTDP-43, infarcts or CAA were significantly associated with either soluble or insoluble FLNA (data not shown).

## Discussion

This study describes the association between AD neuropathology and FLNA levels in post-mortem parietal cortex samples. Results show significant positive associations between most Aβ metrics and insoluble FLNA levels. In contrast, our analyses did not display any significant association between insoluble FLNA levels and tau measurements. Specifically, we found greater insoluble FLNA levels at the ABC intermediate stage as well as in the prodromal AD and ADD stages. We found this to be dependent on the APOE ε4 allele, where noncarriers displayed increased insoluble FLNA levels while ε4 carriers did not. The highest insoluble FLNA average levels, found at the prodromal stage, were mirrored by lower soluble FLNA levels. However, soluble FLNA levels were normal at the ADD stage. These alterations of both insoluble and soluble FLNA levels were found to be excellent predictors of prodromal AD within all subjects with an MCI. Complementary analyses yielded no significant association between insoluble FLNA with presynaptic marker synaptophysin, cholinergic markers ChAT and VAChT, and cognitive scales.

### Insoluble FLNA is associated with amyloid, but not with tau pathology

We found that the insoluble FLNA levels were significantly positively correlated with most Aβ-derived metrics. Associations with the quantifications of insoluble Aβ_42_ and NP from the parietal cortex samples as well as with Aβ-derived Thal staging were of a medium-to large effect size (ρ and R between.391 and.451). In contrast, none of the tau-derived metrics – Braak stages, pTau and total Tau – were found to be significantly associated with insoluble FLNA levels. This was the case despite strong insoluble FLNA associations with general AD progression scales – ABC and AD clinicopathologic stages – as well as between tau and Aβ-derived metrics. Those results indicate that a transition of FLNA to an insoluble form occurs over the course of AD with a trajectory similar to the progression of Aβ neuropathology. However, this loss of solubility is unlikely to be causally linked with tau neuropathological mechanisms, while it may be specifically associated with Aβ mechanisms. A similar phenomenon was observed with U1-70k, another protein that colocalizes well with tau aggregates, but its accumulation is not well correlated with tau levels (Diner et al., 2014). We hypothesize that FLNA insolubility and tau hyperphosphorylation and aggregation are distinct events influenced by Aβ-borne FLNA abnormalities. Wang et al. (2012) identified that the normal function of FLNA is altered in AD, detaching itself from actin filaments and enabling Aβ_42_ to activate neuroinflammation and tau hyperphosphorylation pathways. The same research team attributed this change of function to an alteration of the structure of FLNA caused by Aβ_42_, with the vast majority of FLNA found in the post-mortem AD brain being in its altered state (Burns and Wang, 2017; Wang et al., 2017). Following its structural alteration due to Aβ, we propose that FLNA would be more prone to a transition towards an insoluble state.

### Highest FLNA levels at the intermediate stages of AD

Insoluble FLNA levels were significantly higher at the intermediate stage of AD neuropathologic changes on the ABC scale when compared to the “not” stage. Similarly, insoluble FLNA levels were higher in both the prodromal AD and ADD stages when compared to the non-AD subjects. Insoluble FLNA levels reached their highest values at the mid-stages of Thal (stage 3; Figure 1C), ABC (intermediate stage; Figure 3A) and AD clinicopathologic stages (prodromal AD; Figure 3B), with no further increase in later stages. In addition, soluble FLNA levels were specifically lower in prodromal AD when compared to all other AD clinicopathologic stages. Because of this, we hypothesize that a compensatory mechanism might be engaged at later stages of AD, allowing the stabilization of insoluble FLNA, which would result in an increase of soluble FLNA over time. A second explanation may be that FLNA-stricken neurons have degenerated at the ADD stages, driving down insoluble FLNA levels. FLNA protein levels are tightly linked to dendritic morphology. The transition of FLNA to an insoluble state could be a compensatory mechanism to limit dendritic remodeling, which is observed at early stages of AD (Scheibel and Tomiyasu, 1978; Masliah et al., 1991). At later stages of AD, a transition from insoluble to soluble FLNA could take place as a means as an attempt for reestablishing normal dendritic morphology.

A previous study by Tsujikawa et al. (2022) did not find any AD-associated changes of FLNA levels. However, the sample size of the current study is three and a half times the size of the one that was used in this study (N = 57 vs. N = 16). In addition, the authors did not consider “intermediate” AD neuropathologic change as sufficient for an AD neuropathological diagnostic, with one of the five control subjects likely reaching the criteria for this stage (Braak stage III, moderate CERAD stage, Thal stage undisclosed). From the present study, it appears that this intermediate stage needs to be considered when examining FLNA alterations in post-mortem brain.

In a previous study, a co-localization between FLNA and NFTs was noted (Feuillette et al., 2010). However, such a co-localization did not imply that there was a link between FLNA and tau aggregation. Such a link was demonstrated in a recent study reporting that FLNA contributed to tau aggregation in PSP. Based on this, we examined whether there was a correlation between FLNA and tau insolubility during the progression of AD. No correlation was found indicating that in AD, FLNA and tau aggregation could be two independent processes. This does not exclude the possibility that FLNA could contribute to the increase of tau protein levels and phosphorylation as observed in cellular and animal models. Consistent with this, a similar increase of FLNA and tau protein levels was noted in the whole homogenates of AD hippocampus (Hondius et al., 2016). All the above observations could also indicate that FLNA needs co-factors for inducing tau aggregation, which could vary in different brain regions affected by tau pathology in tauopathies. Such a possibility is supported by the fact that the composition of tau isoforms and the structure of tau filaments vary in tauopathies. For example, tau filaments in AD composed of the 6 tau isoforms are conformationally distinct from tau filaments composed of 4R tau isoforms in PSP.

Insoluble FLNA trajectory was, however, heavily affected by the APOE genotype. APOE ε4 carriers across all clinicopathologic stages of AD displayed similar insoluble FLNA levels to those of participants without AD (10% ε4 carriers). Noncarriers at the ADD and prodromal AD stages, however, displayed the highest amounts insoluble FLNA. Therefore, it may be possible that APOE ε4 enables an AD pathological pathway that does not involve FLNA. This shows that FLNA alteration might not be a necessary condition for the induction of tau neuropathology in APOE ε4 carriers. This highlights that APOE ε4 noncarriers may experience larger FLNA alterations than were estimated in the whole sample.

### FLNA is an effective marker to identify prodromal AD within the MCI

We found that both soluble and insoluble FLNA levels were excellent predictors of prodromal AD from within all MCI participants. The ratio of soluble over insoluble FLNA yielded similar results and did not appear to generate any additional feature. However, only insoluble FLNA levels were an adequate predictor for all-stages AD from within the whole sample. In contrast, NP count and insoluble Aβ_42_ were excellent predictors of AD at all stages, including the identification of preclinical AD from all subjects with NCI. Both total Tau and pTau levels were either adequate or poor predictors of ABC-determined AD. However, we suspect that, as previously mentioned, the sampling region was a determining factor for the lack of effectiveness of tau quantification as a predictor of early stages of AD. In addition, we must acknowledge that the vast majority of subjects with at least an “intermediate” level of neuropathological changes at the ABC staging system display significant NP and Aβ plaque distribution throughout the cortex (Montine et al., 2012). This means that using these measures for the identification of AD is biased, as NP and Aβ plaque detection is used in the participant categorization system. In addition, as mentioned previously, we identified that APOE ε4 carriers did not display AD-associated alterations of FLNA levels. Despite this, FLNA performed well as a predictor of AD from the prodromal stage onward. This hints that, in APOE ε4 noncarriers, FLNA alterations could be a hallmark of AD along with Aβ and tau alterations. The elevated state of insoluble FLNA at both ADD and prodromal AD as well as the lowered levels of soluble FLNA specific to the prodromal AD stage hints that FLNA-derived biomarkers have excellent potential for the identification of AD from symptom onset. Conversely, FLNA was not useful in identifying preclinical subjects from the NCI despite harboring AD neuropathologic changes at levels similar to the prodromal AD. This hints that normal FLNA levels at the preclinical stage of AD might be associated with mechanisms of resilience, maintaining cognitive function despite the progression of AD neuropathology.

### FLNA is not specifically associated with neurodegeneration and cognitive decline

While FLNA alterations coincides with the prodromal AD stage, where cognitive decline and neurodegeneration is significant (Scheff et al., 2006; Vanhaute et al., 2020), insoluble FLNA was not significantly associated with synaptophysin, VAChT, ChAT nor with any cognitive measures. This shows that, while the increased insoluble FLNA levels are a potential hallmark of prodromal AD, it is not a marker of the clinicopathologic progression. We hypothesize that this may be due to the tight regulation of FLNA levels, where both increases and decreases may result in synaptic changes (Zhang et al., 2014). While this may eventually lead to significant synaptic loss and cognitive decline, the latter are indicative of a structural, cumulative change as opposed to the putatively dynamic nature of FLNA levels. In addition, neurodegeneration and cognitive decline are much more pronounced at the ADD stage (DeKosky et al., 1996; Coleman and Yao, 2003). In contrast, insoluble FLNA levels in ADD are not further increased from prodromal AD levels, further explaining why FLNA failed to predict cognitive decline and neurodegeneration.

### Limitations and further studies

Based on the literature and our data, it is possible that FLNA could differently contribute to tau pathology depending on the brain region. Therefore, the conclusions drawn for the parietal cortex might not be applicable to all regions affected by tau pathology in the AD brain. As such, this study specifically shows that tau pathology in the parietal cortex is not correlated with AD-associated FLNA alterations from this region. Additionally, while interpreting the results from this study, one must bear in mind that: (1) the criteria used for the definition of MCI are quite stringent; results may differ when using the most frequent single-domain MCI criteria (Petersen et al., 2014, 2018) and (2) while the same terminology from the NIA-AA’s research framework was used, the biological definition is different. Therefore, participants labeled as prodromal AD in our study may not have received the same diagnosis in an *in vivo* biomarker study.

Previous studies have confirmed that FLNA is detectable in the plasma (Alper et al., 2009). The next steps for FLNA characterization in AD would be to investigate the effect of AD on its presence in the CSF and plasma, which could become *in vivo* biomarkers. These may offer new insight over AD neuropathological processes and may be useful in AD clinical trial. In addition, replicating this study in a larger sample could allow to identify finer features of the trajectory of FLNA throughout the progression of AD as well as identifying potential associations with neurodegeneration.

### Conclusion

In this study, we have found associations between Aβ-derived metrics and insoluble FLNA, hinting that FLNA accumulation are a downstream effect of Aβ neuropathology. In contrast, tau-derived metrics were not associated with insoluble FLNA. We have also described a possible trajectory of FLNA level changes in AD. Insoluble FLNA peaked at the intermediary stages of AD, with heightened levels also found at the later AD stages. These characteristics were more pronounced in APOE ε4 noncarriers. In contrast, soluble FLNA levels specifically dipped at the prodromal AD stage, with all other stages displaying similar soluble FLNA levels. Thanks to the unique features of both soluble and insoluble FLNA at the prodromal AD stages, both metrics were excellent predictors of AD within the MCI subjects. As such, our results show that, in APOE ε4 noncarriers, FLNA alterations may be a neuropathological hallmark of AD, which may contribute to the transition from a preclinical to a prodromal stage of AD.

## Data availability statement

All requests for raw and analyzed data and materials will be promptly reviewed by RUMC to obtain a third-party sharing agreement. Anonymized data will be shared upon request to the study’s senior author from a qualified academic investigator for the sole purpose of replicating the procedures and results presented in this article. Any data and materials that can be shared will be released via a material transfer agreement. Data are not publicly available due to information that could compromise the privacy of research participants.

## Ethics statement

The studies involving human participants were reviewed and approved by Institutional Review Board of Rush University Medical Center. The patients/participants provided their written informed consent to participate in this study.

## Author contributions

EA, SL, FC, and NL developed the research project. CT performed the brain sample analyses. DB provided the brain samples. All authors contributed to the manuscript and approved the final submitted version.

## Funding

This study was provided by the Canadian Institutes of Health Research (CIHR) to FC (MOP 125930 and PJT 168927) and to NL (PTJ-155993), as well as by CRCHUM internal funds to NL. ROS is supported by National Institutes of Health (NIH) grants (P30AG10161, P30AG72975, and R01AG15819). ROS resources can be requested at www.radc.rush.edu. EA held a Vanier Canada graduate scholarship. FC is a Fonds de recherche du Québec–Santé (FRQS) senior research scholar.

## Conflict of interest

The authors declare that the research was conducted in the absence of any commercial or financial relationships that could be construed as a potential conflict of interest.

## Publisher’s note

All claims expressed in this article are solely those of the authors and do not necessarily represent those of their affiliated organizations, or those of the publisher, the editors and the reviewers. Any product that may be evaluated in this article, or claim that may be made by its manufacturer, is not guaranteed or endorsed by the publisher.

## Supplementary material

The Supplementary material for this article can be found online at: https://www.frontiersin.org/articles/10.3389/fnagi.2022.1038343/full#supplementary-material
