# Supplementary material for: Evidence of Filamin A loss of solubility at the prodromal stage of neuropathologically-defined Alzheimer’s disease
Source: Front Aging Neurosci. 2022 Nov 24;14:1038343. doi: 10.3389/fnagi.2022.1038343 (PMC9730531; doi:10.3389/fnagi.2022.1038343)

**Supplementary table 1: Summary of statistical tests for all partial correlations adjusted for age, sex and study batch between biological measures and AD stages.** Insoluble FLNA, Aβ_42_, neuritic plaques, total tau and phosphorylated tau were residualized for age, sex, and study batch. Synaptophysin, VAChT and ChAT were residualized for age and sex since this data was not available for the second batch.

| Dependent variable | Independent variable | Raw p-value | FDR-adjusted p-value | β/ρ |
| --- | --- | --- | --- | --- |
| Insoluble Filamin A | Aβ_42_ | 0.006 | 0.036* | 0.406 |
|  | Neuritic plaques (log transformed) | 0.017 | 0.042* | 0.353 |
|  | Total tau | 0.850 | 0.927 | 0.028 |
|  | Phosphorylated tau | 0.978 | 0.978 | -0.004 |
|  | Synaptophysin | 0.275 | 0.367 | 0.219 |
|  | VAChT | 0.146 | 0.218 | -0.28 |
|  | ChAT | 0.088 | 0.176 | -0.335 |
|  | CERAD scale | 0.122 | 0.209 | 0.207 |
|  | Thal stages | 0.016 | 0.042* | 0.318 |
|  | BRAAK stages | 0.440 | 0.528 | 0.104 |
|  | ABC scale | 0.017 | 0.042* | 0.316 |
|  | Clinicopathological stages of AD | 0.003 | 0.036* | 0.386 |
| Aβ_42_ | Total tau | 0.004 |  | 0.401 |
|  | Phosphorylated tau | 0.017 |  | 0.340 |
| Neuritic plaques | Total tau | 0.0003 |  | 0.494 |
|  | Phosphorylated tau | 0.0001 |  | 0.524 |
| Braak stages | CERAD scale | <0.0001 |  | 0.503 |
|  | Thal stages | <0.0001 |  | 0.596 |

**Supplementary table 2: Receiver-operating characteristics analyses for predicting AD neuropathology (intermediate or high neuropathologic change on the ABC scale) in the whole sample, the MCI subgroup or the NCI subgroup.** Predictors were residualized for age, sex and study batch.

| Samples | Predictors | Specificity | Sensitivity | PPV | AUC | Raw p-value | FDR-adjusted p-value |
| --- | --- | --- | --- | --- | --- | --- | --- |
| Whole sample (N=57) | Insoluble Filamin A | 0.650 | 0.838 | 0.816 | 0.727 | 0.004 | 0.040* |
|  | Soluble Filamin A | 0.800 | 0.514 | 0.826 | 0.620 | 0.140 | 0.185 |
|  | Soluble/insoluble Filamin A ratio | 0.700 | 0.702 | 0.813 | 0.684 | 0.023 | 0.041* |
|  | Total tau | 0.950 | 0.432 | 0.941 | 0.682 | 0.024 |  |
|  | Phosphorylated tau | 0.950 | 0.532 | 0.941 | 0.716 | 0.007 |  |
|  | Aβ_42_ | 0.750 | 0.891 | 0.868 | 0.846 | <0.0001 |  |
|  | Neuritic plaques | 0.900 | 0.946 | 0.946 | 0.949 | <0.0001 |  |
| Mild cognitively impaired (N=19) | Insoluble Filamin A | 0.875 | 0.818 | 0.889 | 0.818 | 0.020 | 0.041* |
|  | Soluble Filamin A | 0.625 | 0.727 | 0.786 | 0.830 | 0.016 | 0.041* |
|  | Soluble/insoluble Filamin A ratio | 0.875 | 0.818 | 0.900 | 0.852 | 0.009 | 0.041* |
|  | Total tau | 0.875 | 0.636 | 0.875 | 0.761 | 0.062 |  |
|  | Phosphorylated tau | 0.375 | 1 | 0.688 | 0.739 | 0.091 |  |
|  | Aβ_42_ | 0.750 | 1 | 0.846 | 0.875 | 0.005 |  |
|  | Neuritic plaques | 1 | 0.909 | 1 | 0.966 | 0.0002 |  |
| Not cognitively impaired (N=19) | Insoluble Filamin A | 1 | 0.222 | 1 | 0.556 | 0.720 | 0.905 |
|  | Soluble Filamin A | 0.800 | 0.556 | 0.714 | 0.589 | 0.549 | 0.559 |
|  | Soluble/insoluble Filamin A ratio | 1 | 0.333 | 1 | 0.600 | 0.497 | 0.559 |
|  | Total tau | 0.700 | 0.556 | 0.625 | 0.522 | 0.905 |  |
|  | Phosphorylated tau | 0.600 | 0.556 | 0.556 | 0.444 | 0.720 |  |
|  | Aβ_42_ | 0.800 | 0.889 | 0.800 | 0.800 | 0.028 |  |
|  | Neuritic plaques | 0.900 | 0.889 | 0.889 | 0.900 | 0.002 |  |
| *: FDR-adjusted p<0.05 | | | | | | | |

**Supplementary table 3: Semi-partial correlations of insoluble FLNA, Aβ_42_, neuritic plaques, total tau and phosphorylated tau with cognitive assessments.** Biological measures were corrected for APOE ε4 carrier status, age, sex, and study batch, while cognitive measures were corrected for APOE ε4 carrier status, age, sex, and years of education.

| Dependent measure | Indicator | Insoluble Filamin A | Phosphorylated Tau | Total Tau | Aβ_42_ | Neuritic plaques |
| --- | --- | --- | --- | --- | --- | --- |
| Episodic memory CS | R | -0.018 | -0.514 | -0.546 | -0.429 | -0.458 |
|  | Raw p-value | 0.895 | <0.0001**** | <0.0001**** | 0.001*** | 0.0006*** |
| Semantic memory CS | R | 0.099 | -0.407 | -0.468 | -0.303 | -0.402 |
|  | Raw p-value | 0.469 | 0.002** | 0.0003*** | 0.023* | 0.002** |
| Working memory CS | R | 0.042 | -0.393 | -0.445 | -0.299 | -0.304 |
|  | Raw p-value | 0.760 | 0.003** | 0.0008*** | 0.027* | 0.025* |
| Visuospatial CS | R | 0.045 | -0.211 | -0.269 | -0.335 | -0.286 |
|  | Raw p-value | 0.731 | 0.102 | 0.038* | 0.008** | 0.025* |
| Processing speed CS | R | -0.198 | -0.270 | -0.383 | -0.330 | -0.347 |
|  | Raw p-value | 0.134 | 0.040* | 0.003** | 0.011* | 0.007** |
| General cognition CS | R | -0.065 | -0.494 | -0.555 | -0.488 | -0.476 |
|  | Raw p-value | 0.634 | 0.0001*** | <0.0001***** | 0.0001**** | 0.0002*** |
| Last MMSE score | R | -0.225 | -0.436 | -0.525 | -0.604 | -0.417 |
|  | Raw p-value | 0.093 | 0.0007*** | <0.0001**** | <0.0001****** | 0.001*** |
| CS: composite score, *: raw p<0.05, **: raw p<0.01, ***: raw p<0.001, ****: raw p<0.0001, *****: raw p<0.00001, ******: raw p<0.000001 | | | | | | |

**Supplementary figure 1: Insoluble total tau (itTau) association with ABC scores and Braak stages.** Insoluble tau levels are specifically heightened at the late AD neuropathological stages. Total tau was corrected for age, sex, and study batch. Predicted insoluble total tau values are calculated by adding the mean to the residual values from age, sex and study batch linear regression. ***: p < 0.001


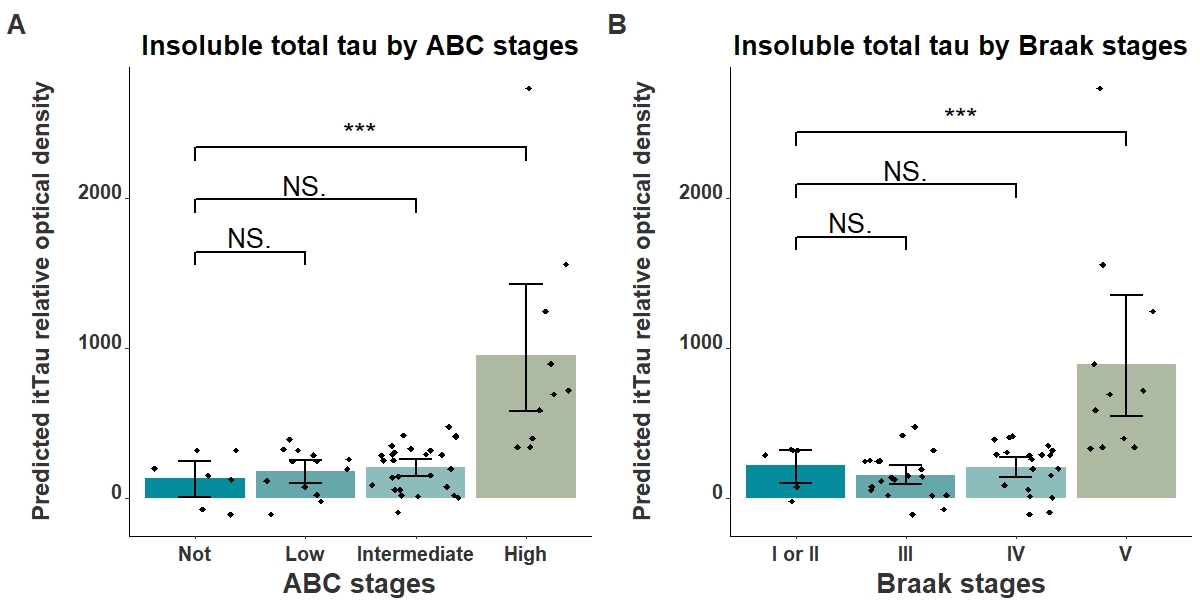


**Supplementary figure 2: Correlations between Aβ-derived measures and tau-derived measures.** Predicted insoluble Aβ_42_ (iAβ_42_), total tau (itTau) and phosphorylated tau (ipTau), as well as neuritic plaques (NP) values are calculated by adding the mean to the residual values from age, sex and study batch linear regression.


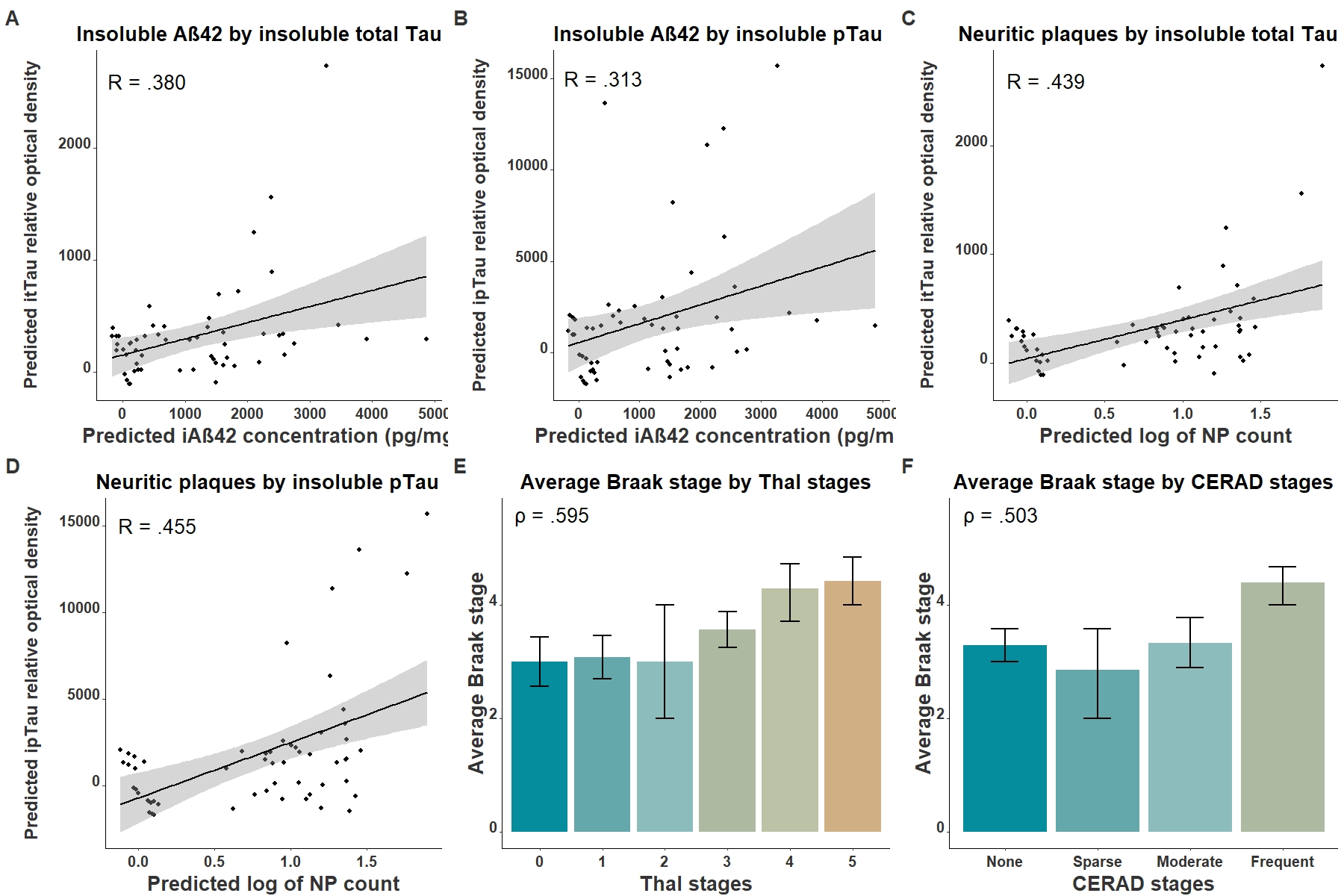

Supplement: Supplementary file 1 [file Data_Sheet_1.docx]
